# Ways to Eliminate Viral Hepatitis as a Global Health Threat

**DOI:** 10.3390/v14071554

**Published:** 2022-07-16

**Authors:** Robert Flisiak, Dorota Zarębska-Michaluk, Marta Flisiak-Jackiewicz

**Affiliations:** 1 Department of Infectious Diseases and Hepatology, Medical University of Białystok, 15-540 Białystok, Poland; 2 Department of Infectious Diseases, Jan Kochanowski University, 25-317 Kielce, Poland; dorota1010@tlen.pl; 3 Department of Pediatrics, Gastroenterology, Hepatology, Nutrition and Allergology, Medical University of Bialystok, 15-247 Bialystok, Poland; m_flisiak@op.pl

## 1. Introduction

Hepatitis B (HBV) and C (HCV) have been recognized by the World Health Organization.

(WHO) as one of the major public health problems worldwide. Hepatotropic viruses are estimated to be responsible for the deaths of more than one million people annually, making their mortality comparable to human immunodeficiency virus infections, tuberculosis and malaria. According to recent epidemiological reports, 71 million people with HCV live in the world [1]. The introduction of highly effective and safe direct-acting antivirals (DAAs) has radically changed the landscape of HCV treatment [2,3]. In 2016, the WHO initiated a plan to eliminate viral hepatitis as a public health threat by 2030 [4]. Unfortunately, the implementation of this ambitious plan was disrupted by the COVID-19 pandemic, which resulted in a reduction in the number of screening tests performed and the number of patients treated for viral hepatitis.

This Special Issue of *Viruses* “Ways to eliminate viral hepatitis as a global health threat” aims to provide updated information on the efforts and the opportunities to achieve the WHO target. The issue contains twelve original and review articles covering the description of the epidemiological situation of HCV infections in various countries, the advancements in screening programs, problems related to linkage to care, a discussion of modern diagnostic possibilities and, finally, the challenges of therapy. This Special Issue shows the current state of HCV elimination in various parts of the world, but also the best possible ways of proceeding to achieve the goals set by the WHO.

## 2. Testing and Linkage to Care

Jonas et al. [5] retrospectively analyzed alternative treatment methods available in three US states in the search for the optimal method of testing and then linkage to care, leading to effective treatment of HCV infections. Out of over 130,000 respondents, more patients were tested through a coordinator-supported, standardized testing pathway completing the necessary testing steps, in less time, compared to usual care. This indicates that it is advisable to create dedicated HCV infection elimination programs that are planned and coordinated at various levels. In countries where this is impossible, usually due to the reluctance of central or local authorities, it is justified to screen patients admitted to hospitals for various reasons, which is able to provide quick diagnosis and start treatment of HCV infection. The legitimacy of such a procedure was confirmed by Rosato et al. [6] on the basis of the results of a program implemented at the Evangelical Hospital Betania of Naples. This program allowed for the detection of 91 infected people among 12,665 tested subjects, 96% of whom underwent treatment and obtained a sustained virologic response.

The analysis of the epidemiological situation in eight Central European countries, based on expert opinions, showed the scale of the region’s problems in the elimination of HCV. According to the estimates provided, nearly 400,000 inhabitants of this region are infected with HCV, usually without realizing it. Despite the sufficient availability of DAA treatment, the number of patients treated has decreased in recent years compared to the 2017–2019 period. This was mostly due to the COVID-19 pandemic, limiting the possibility of implementing screening programs, which were not supported by government institutions regardless. None of the analyzed countries see a possibility to achieve the goals set by the WHO of eliminating viral hepatitis, as long as the barriers, such as the lack of political will and screening programs, are not removed [7]. As shown by the results of a detailed analysis in Lithuania, as many as 41% of cirrhosis patients, 50% of liver cancer patients and 37% of patients requiring liver transplantation were infected with HCV [8]. However, in light of another work published as part of our Special Issue, the scale of problems related to HCV infections is much greater in Russia, where the most important barriers to HCV elimination are high prevalence of infection, low access to treatment, especially in populations at high risk of HCV infections, and centralization of the healthcare system, making linkage to care difficult [9].

## 3. Screening and Treatment in Prisons

A particular challenge, even in countries that have managed to introduce national HCV elimination programs, remain the groups at increased risk of infection, a category to which prisoners belong. The frequency of infections in this group in Central European countries is many times higher than that found in the general population, but precise, current data are not known due to the lack of scheduled screening tests. No country in the region has implemented a national program for the eradication of HCV in prisons, which requires testing to be offered to all inmates and, if infection is confirmed, to provide optimal treatment. The main reason is a lack of will at the governmental and prison levels [7]. Despite this, there are plans for coordinated action in individual countries, as exemplified by a Hungarian study in which 28% of inmates in 2018–2019 were tested for HCV and 82% of them received DAA treatment [10]. Undoubtedly, Australia has the greatest experience in the implementation of the HCV infection control program in prisons, where in 2020 as much as 37% of all DAA treatments were conducted in prisoners [11]. As a result of the implementation of the multi-annual program, the percentage of people infected with HCV in prisons decreased by nearly a half. The article by Winter et al. [11] published in our Special Issue is a kind of guide for other countries, especially those where there is strong resistance among the prison authorities against introducing HCV infection elimination programs. Additionally, the authors point to future actions involving the intensification of prevention, a further increase in the number of tests, and finally, the continuation of care for inmates after completing their sentence.

## 4. Diagnostics

One of the major problems in the elimination of HCV infection is the loss of patients between screening and treatment. This is due to the need for several patient visits in order to: detect anti-HCV antibodies, confirm the activity of the infection by HCV RNA testing, perform other tests required by the healthcare system to reimburse the therapy, and finally start treatment. One way to reduce the number of these steps has been tested and published by Petroff et al. [12]. The authors compared standard procedures with point-of-care assays enabling the detection of HCV-RNA, using the GeneXpert instrument to obtain a result within 60 min and make a diagnosis, with possible initiation of treatment in one visit. Primary care physicians in an additional survey rated the use of this technique as bad, satisfactory, or good in 6%, 13%, and 81% of cases, respectively.

Recently, pangenotypic therapy, which has been dominant in the treatment of HCV infections, not only made viral genotyping redundant, but also the assessment of disease advancement by assessing fibrosis lost its importance. However, despite the lack of significant differences in the treatment lengths and methods, the knowledge of the stage of liver disease may be helpful in predicting the effectiveness of treatment, especially the need for further monitoring for the risk of developing hepatocellular carcinoma. The analysis of currently available methods of assessing fibrosis is presented in the paper by Cardoso et al. [13].

## 5. Treatment of Difficult and Young

Despite the high efficacy of the currently available therapeutic regimens, there is still a small group of patients not responding to treatment. Their identification is crucial due to the possibility of taking action for increasing the adherence of these patients and further observation for possible rescue therapy. Selecting such a group requires a study involving a large group of patients. Pabjan et al. [14], analyzing a group of nearly a thousand patients in detail, showed that the factors lowering the chance of effective DAA treatment are: male sex, genotype 3 infection, cirrhosis and previous therapy failures. Patients with such characteristics require special supervision during and after treatment.

DAA-based treatment became available to children and adolescents with the delay typical of all new therapies requiring additional follow-up in this age group after registration in adults. Conducting clinical trials, and, more importantly, real-world experience, on the use of drugs in children is additionally hampered by the much lower incidence of HCV infections in those under the age of 18 in Europe. It is particularly difficult in the case of children with advanced fibrosis and cirrhosis, and this group of children was successfully treated with ledipasvir/sofosbuvir (LDV/SOF) in the study by Pokorska-Śpiewak et al. [15]. When new medicines are used in children, there is always a concern about an impact on their development. Another study published in our Special Issue proved that LDV/SOF treatment did not adversely affect the growth of treated children. A relationship was observed between a high baseline BMI and the advancement of fibrosis and steatosis, which, however, did not affect the effectiveness of the therapy [16].

## 6. Conclusions

The variety of topics included in the Special Issue “Ways to eliminate viral hepatitis as a global health threat” made it possible to trace the pathways leading to a reduction in or even elimination of HCV infections as a serious global health problem in the future. The multitude of problems and the presented patterns of solving them indicate the advisability of further research and publications on this topic. This also justifies the preparation of the second edition of a Special Issue in *Viruses* devoted to the elimination of viral hepatitis, perhaps taking into account the topic of HBV infections, which was unfortunately omitted in the first edition.
